# Changes in Inflammatory Cytokines and Irisin in Response to High Intensity Swimming in Adolescent versus Adult Male Swimmers

**DOI:** 10.3390/sports8120157

**Published:** 2020-12-01

**Authors:** Malcolm Sanderson, Brandon J. McKinlay, Alexandros Theocharidis, Rozalia Kouvelioti, Bareket Falk, Panagiota Klentrou

**Affiliations:** 1 Department of Kinesiology, Brock University, St. Catharines, ON L2S 3A1, Canada; ms16ot@brocku.ca (M.S.); brandon.mckinlay@brocku.ca (B.J.M.); theocharidisalex@gmail.com (A.T.); rkouvelioti@brocku.ca (R.K.); bfalk@brocku.ca (B.F.); 2 Centre for Bone and Muscle Health, Brock University, St. Catharines, ON L2S 3A1, Canada

**Keywords:** boys, men, athletes, high-intensity interval exercise, inflammation

## Abstract

Swimming is a popular youth sport that is considered beneficial for cardiovascular fitness. However, the potential inflammatory outcomes of high intensity swimming in younger swimmers are unclear, as is the response of irisin, a myokine released during exercise with anti-inflammatory properties. This study compared the plasma concentrations of interleukins 1-beta (IL-1β), 6 (IL-6), 10 (IL-10), tumor necrosis factor alpha (TNF-α) and irisin in response to intense swimming between adolescent and adult male swimmers. Thirty-two swimmers (16 adolescents, 14 ± 1 years; 16 adults, 21.5 ± 3.1 years) completed a high intensity interval swimming trial. At rest, only TNF-α was higher (33%, *p* < 0.05) in adolescents compared with adults. There was an overall significant increase in IL-1β from pre- to post-swimming (3% in adolescents, 24% in adults), but no significant interaction. IL-10 significantly increased in both groups (+34% in adolescents, +56% in adults). IL-6 and TNF-α increased significantly (+32% and +26%, respectively) in adults, but not in adolescents (+2% and −9%, respectively). Adults showed a small, but significant decrease in irisin (−5%), with no change in adolescents. The lack of an IL-6, TNF-α and irisin response to intense swimming in adolescent swimmers may suggest a blunted inflammatory and myokine response following high intensity exercise in trained youth.

## 1. Introduction

Intense exercise can significantly influence several inflammatory markers, while the magnitude and direction of changes in specific cytokines, may be mediated by the mode and intensity of the activity, as well as the age and training status of participants [1,2]. For example, although children recover faster than adults from intense exercise, not just in terms of performance, but also in terms of physiological responses [3,4], there is no consistent evidence of age-related differences in the inflammatory response to intense exercise. In recent years, there has been increasing interest in studying the inflammatory response after various forms of intense exercise in both adults and children involved in high performance sports, due to the potential that repetitive bouts of high intensity exercise with little recovery time may lead to chronic low-grade inflammation [1].

Interleukin 1-beta (IL-1β), interleukin 6 (IL-6), interleukin 10 (IL-10), and tumor necrosis factor alpha (TNF-α) are known pro-inflammatory cytokines that are released in the early stages of the inflammation process in response to muscle injury to initiate the muscle regeneration process [5]. IL-10 is an anti-inflammatory cytokine released later to repress the pro-inflammatory effects and to support potential differentiation of muscle stem cells [5]. In young, non-athletic men, previous research has observed increases in TNF-α [6,7,8], IL-6 [6,7,8,9], and IL-10 [6,7,8], but not IL-1β [8], following various exercise protocols compared to pre-exercise levels. However, there has been much less research regarding the cytokine response to exercise in children and adolescents, with studies reporting mixed results of both increases [10,11,12], and no changes post-exercise [9]. Although the intensity of these studies ranges from moderate to intense, and irrespective of the limited evidence, there is agreement that muscle damage associated with exercise, which produces an inflammatory response, may be less in children than in adults [13,14]. However, whether this blunted inflammatory response is also true for youth athletes is unclear. In addition, irisin is a myokine secreted by skeletal muscles during exercise, which induces browning of white adipose tissue [15]. Recent evidence also suggests that irisin may also have anti-inflammatory properties, leading to the reduction of secretion of inflammatory cytokines like ΙL-6 and TNF-α [16]. Irisin has been shown to immediately increase post-exercise in both adults [17,18,19,20] and children [20,21] while its response to exercise is intensity-dependent, with larger post-exercise increases following higher intensity exercise [17].

Most available data on the inflammatory response to exercise relate to prolonged, moderate exercise. However, the increase in cytokine levels following repeated bouts of intense exercise, may be attenuated compared with the response following the initial bout [22]. It is possible that the anti-inflammatory cytokine IL-10 and irisin are related to the attenuated response following repeated bouts. In addition, there are no studies that have directly compared the acute post-exercise differences in cytokine and irisin responses to the same intense exercise protocol in adolescent and adult athletes. Swimming, which is associated with metabolic, musculoskeletal, and cardiorespiratory benefits [23], is a popular sport among youth. It is, however, different from other land-based sports, in that is predominantly concentric and low impact, and has been shown to cause different biochemical reactions than land-based sports [24]. Indeed, swimming generally induces less muscle damage, and potentially a lower inflammatory response, compared to eccentric activities (e.g., resistance or plyometric exercise), which are known to result in considerable inflammation, especially during novel exercise [25]. Thus, the purpose of this study was to examine the age-related differences in the acute response of anti- and pro-inflammatory cytokines (IL-1β, IL-6, IL-10, and TNF-α) and irisin to a high intensity interval swimming trial in adolescent and adult male swimmers.

## 2. Materials and Methods

### 2.1. Participants

Participants in the current study included 16 adolescent (11–15 years old) and 16 adult (18–30 years old) male swimmers, who trained and competed for at least four years, were free of injuries and any medical condition that prevented them from participating and were not taking medications or nutritional supplements. Swimmers were recruited from two varsity teams and five local swim clubs in Southern Ontario as part of a larger study designed to examine the effect of post-exercise whey protein consumption on subsequent exercise performance, muscle damage, and inflammation [26]. Data presented in this study are a secondary analyses of blood samples taken before the protein/carbohydrate supplementation protocol and solely focus on acute responses to intense swimming.

All participants and their parents/guardians were provided a thorough explanation of the study’s purpose, procedures, and potential risks. Consent was obtained from all participants and their respective parent/guardian where applicable. The study procedures and secondary analysis presented herein were cleared by the Research Ethics Boards of Brock University (REB# 16-279 and 18-296, respectively) and the Canadian Sports Institute of Ontario (REB# 2017-01).

### 2.2. Study Design

The study employed a parallel cross-sectional design which included two visits. The first visit was an information session, which also included anthropometric measurements. Details of the measurements can be found in McKinlay et al. [26]. In brief, body fat and body mass (kg) were collected using bioelectric impedance analysis (InBody520; BioSpace Co, Ltd., Madison, MI, USA). Participants self-hydrated ad libitum prior to the visit and were asked to void before body measurements were performed. Seated and standing height were recorded with a stadiometer (SECA-217; CAN), and were used to assess somatic maturity, indicated as years from age of peak height velocity [27].

Between visits 1 and 2, participants were instructed to refrain from any exercise for a minimum of 48 h prior to visit 2. The second visit included the swimming trial and two blood samples: one fasted (i.e., after an overnight fast of 10–12 h), a resting (i.e., pre-swimming) sample collected at 06:00 h, and one post-swimming sample collected within 15 min following swimming. Both the swimming trial and the blood draws are described below. Immediately after the resting blood sample and ~1 h before swimming, participants were provided a standardized breakfast of ~300–500 kcal (depending on body mass), which included a granola bar, muffin, fruit (banana or apple) and a juice box or water. Details of the ingredients of the breakfast can be found in McKinlay et al. [26].

### 2.3. High Intensity Swimming Trial

The swimming trial commenced ~1.5 h after the pre-exercise blood draw (~1 h postprandial) and it was designed for the purposes of the larger study to mirror a day of swimming competition. Details of the larger study’s protocol and measurements can be found in McKinlay et al. [26]. Briefly, the trial started with participants performing a warm-up of a 1000 m swim, followed by a maximal 200 m front-crawl swim test. Approximately 5 min following the maximal swim test, participants performed a high intensity interval swimming (HIIS) protocol, which consisted of 5 × 100 m, 5 × 50 m and 5 × 25 m at >90% of each swimmer’s maximum performance time, as calculated from their split time (100 m) during the 200 m maximal swim test, and using a 1:1 work-to-rest-ratio.

### 2.4. Blood Collection and Analysis

In order to control for circadian rhythm, all testing was performed in the morning hours. Blood samples were collected at rest (06:00 h, fasted), and immediately (within 15 min) following the swimming trial (09:00–10:00 h). Venous samples (10 mL) were drawn from the antecubital fossa using a standard venipuncture technique. All samples were centrifuged at 1405× *g* at 4 °C using a benchtop centrifuge for 10 min. Serum and plasma were then aliquoted into pre-labeled Eppendorf tubes to be stored at −80 °C until analysis.

Plasma concentrations of IL-1β, IL-6, IL-10 and TNF-α were measured in duplicates using multiplex magnetic bead kits (Cat. #HSTCMAG-28SK, Milliplex EMD Millipore Corporation, Burlington, MA, USA). The average in-house inter- and intra-assay coefficients of variation (CV) for IL-6, IL-10, IL-1β and TNF-α were 8.1% and 4.3%, 6.5% and 4.3%, 4.6% and 7.3%, 8% and 5.3%, respectively. Irisin was also measured in duplicate using an irisin recombinant assay kit (Cat #EK-067-29, Phoenix Pharmaceuticals Inc., Burlingame, CA, USA). The average in-house inter- and intra-assay CV for irisin were 1.2% and 4%, respectively.

For every blood draw, hematocrit was measured in triplicates by the same investigator using microhematocrit capillary tubes treated with heparin (VWR International, Radnor, PA, USA). Relative plasma volume changes (%Δ*PV*) were calculated using the formula by Van Beaumont [28].

$$\%\Delta PV=\frac{100}{100-Hct1}\times \frac{100\left(Hct1-Hct2\right)}{Hct2}\%$$

where *Hct*1 is hematocrit at baseline, and *Hct*2 is hematocrit at each post-baseline measurement. The %Δ*PV* was then used to adjust the post-swimming plasma concentrations of inflammatory cytokines (IL-6, IL-10, IL-1β, TNF-α) and irisin using the formula: 100+%Δ*PV*/100.

### 2.5. Statistical Analysis

All variables were first checked for normality using the Kolmogorov-Smirnov test; variables were also checked for skewness and kurtosis of ±3. In addition, visual screening of histograms for symmetry was performed. IL-1β, IL-6, IL-10 and irisin concentrations were not normally distributed, and were log transformed for further analysis. Group differences in physical characteristics, resting cytokines and irisin levels were analyzed using independent t-tests. A two-way (group-by-time) ANOVA for repeated measures was used to examine changes in cytokines (IL-1β, IL-6, IL-10, TNF-α) and irisin levels (pre- to post-swimming). In the event of a significant interaction, post-hoc comparisons were performed within each group using paired t-tests. The baseline characteristics are reported as mean ± standard deviation (SD) and cytokine concentrations at rest are reported as mean ± standard error (SE) in descriptive tables. The results of the ANOVA repeated measurements are shown reporting F, *p* and partial η^2^. Effect size for partial η^2^ was then interpreted based on the Cohen criteria: 0.01 = small effect, 0.06 = moderate effect, and 0.14 = large effect. Statistical significance was accepted at *p* < 0.05, which was conducted using SPSS version 26 for Windows (SPSS Inc., Chicago, IL, USA).

## 3. Results

Baseline physical and training characteristics can be found in Table 1 and baseline (i.e., resting) concentrations of cytokines and irisin are presented in Table 2. Inflammatory cytokine levels at rest were not significantly different between groups, with the exception of TNF-α, which was +33% higher (*p* = 0.02) in the adolescent male swimmers compared to the adults (Table 2).

**Figure 1:**
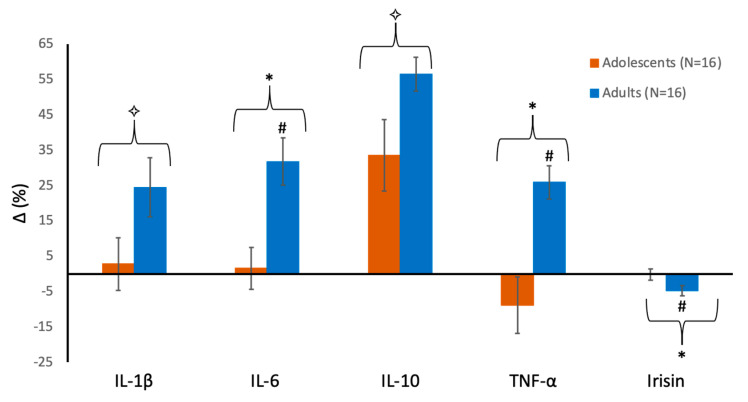
Percent changes (Δ mean ± standard error) from pre- to post-swimming in plasma concentrations of interleukin 1-beta (IL-1β), interleukin 6 (IL-6), interleukin 10 (IL-10), tumor necrosis factor alpha (TNF-α) and irisin in adolescent and adult male swimmers. * denotes a significant difference between groups (group effect, *p* < 0.05). ✧ denotes a significant change from pre- to post-swimming in both groups (time effect, *p* < 0.05). # denotes a significant change from pre- to post-swimming in adults (interaction, *p* < 0.05).

Figure 1 shows the percent changes in the various cytokines from pre- to post-swimming in each group. IL-1β showed a significant effect for time (F = 6.47; *p* = 0.016; partial η^2^ = 0.18), reflecting an overall large increase from pre to post-exercise, with no significant group effect (F = 0.00; *p* = 0.99; partial η^2^ = 0.00) and no significant group-by-time interaction (F = 2.87; *p* = 0.10; partial η^2^ = 0.09). Thus, although IL-1β increased by 3% in adolescents versus 24% in adults, this difference was not statistically significant (Figure 1). IL-6 showed no significant group effect (F = 1.14; *p* = 0.29; partial η^2^ = 0.03), but a significant time effect (F = 14.11; *p* = 0.001; partial η^2^ = 0.32) and a large, significant group-by-time interaction (F = 9.75; *p* = 0.004; partial η^2^ = 0.25), reflecting a significant increase following intense swimming in the adult swimmers (+32%, *p* = 0.002), which was not the case in adolescents (Figure 1). There was a large, significant increase over time for IL-10 (+34% and +56% in boys and men, respectively; F = 40.6; *p* = 0.001; partial η^2^ = 0.57), with no significant group effect (F = 0.49; *p* = 0.49; partial η^2^ = 0.01) or group-by-time interaction (F = 0.99; *p* = 0.33; partial η^2^ = 0.03). For TNF-α, we found no effect for group (F = 2.61; *p* = 0.12; partial η^2^ = 0.08), a significant time effect (F = 9.16; *p* = 0.005; partial η^2^ = 0.23), and a significant group-by-time interaction (F = 16.37; *p* = 0.001; partial η^2^ = 0.35), which reflects a significant increase in the adults (+26%, *p* < 0.001) with a non-significant decrease (−9%, *p* = 0.47) in the adolescents (Figure 1). A significant main effect for time (F = 4.71; *p* = 0.038; partial η^2^ = 0.14), but not for the group (F = 0.29; *p* = 0.59; partial η^2^ = 0.01), and a significant interaction (F = 4.92; *p* = 0.034; partial η^2^ = 0.14) were also found for irisin, reflecting a small yet significant decrease in adults (−5%, *p* = 0.03), with no change in the adolescents (Figure 1).

## 4. Discussion

To our knowledge, this is the first study to directly compare the response of anti- and pro-inflammatory cytokines, and irisin to high intensity interval swimming between two age groups of competitive male swimmers. Previous studies typically examined age-related differences in cytokine and irisin responses to acute exercise in non-athletic populations, who on one hand are accustomed to the specific exercise, but on the other hand are exposed to chronic high intensity, non-eccentric, exercise with little recovery time. Overall, we observed significant exercise-induced increases in all cytokines, and a decrease in irisin, in adults, but these changes were blunted or not evident in the adolescent swimmers, as evidenced by large interaction effects (partial η^2^ ranging from 0.14 to 0.35). Specifically, IL-1β, IL-6, IL-10 and TNF-α significantly increased while irisin decreased from pre- to post-swimming in the adults, but in the adolescents, only the anti-inflammatory IL-10 increased significantly in response to high intensity swimming.

At rest, IL-1β, IL-10 and irisin levels were not significantly different between groups. The only significant age-related difference at rest was in TNF-α, which was 33% higher in the adolescent group compared with adults. Notably, IL-6 was also 47% higher in the adolescents, but this difference was not statistically significant, likely due to its high variability. Studies examining age-related differences in the resting concentrations of inflammatory cytokines are scarce. Timmons et al. [9] also found significantly higher resting levels of IL-6, in their cohort of healthy, non-athletic boys (9.8 ± 0.1 years) compared to 10 young men of similar age to our adult swimmers (22.1 ± 0.5 years). They also found higher resting TNF-α in the boys, albeit the difference was not statistically different. It is possible that the higher resting TNF-α in adolescent swimmers could be indicative of low-grade chronic inflammation due to the high-volume training (~14 h/week) in which they were involved. The average age of these swimmers was 14 years, and in terms of maturity, they were around the age of peak height velocity. Thus, it is possible that the growth spurt poses stress on the immune system, indirectly supported by Timmons et al. [9], who reported higher resting cytokine levels in boys than in men. This growth-induced stress, in combination with the increased physical stress of athletic training may explain the higher resting TNF-α in the adolescent swimmers. On the other hand, the acute inflammatory response to intense exercise was attenuated in the adolescent swimmers compared with the typical exercise-induced increases we saw in adults, which suggests that single intense bouts of exercise may be immunoprotective, while the chronic accumulation of multiple intense training sessions with little recovery time in between may lead to low-grade inflammation in adolescents. Indeed, the resting levels of both IL-6 and TNF-α were substantially higher in our group of young swimmers compared with the non-athletic boys in Timmons et al. [9], which could be attributed to the younger age of their group, or to a low-grade inflammation in our swimmers due to intense training. In a previous study, we monitored elite female rowers across a training year, and showed that resting TNF-α, as well as IL-1β and IL-6, fluctuated in accordance with training load, with circulating concentrations being highest during periods of high training load and lowest during tapering [29].

Our post-swimming results in the adult group of swimmers are in agreement with previous studies examining cytokine response in men to various forms of exercise, generally showing an increase from pre- to immediately post-exercise regardless of exercise modality [6,7,8,9]. Specifically, Zaldivar et al. [7] reported an 80–100% increase in IL-6 from pre- to immediately post-exercise in young men (24.8 ± 0.8 years old) performing a 30-minute bout of exercise on a cycle ergometer at ~80% of their VO_2_ max. In our study, the young men had a 49% increase in IL-6 concentration immediately after the intense swimming trial. However, the study by Zaldivar et al. [7] excluded competitive athletes, so that the exercise regime was novel for participants, which would produce a more robust metabolic stress and immune response. In contrast, our participants were adapted to intense swimming, and chronic exercise training can lead to a blunted cytokine response [30]. In addition, Zaldivar et al. [7] did not adjust for plasma volume changes while a recent study, which did account for plasma volume changes, reported much lower, significant increases in IL-1β, IL-6, IL-10 and TNF-α 5 min and up to 60 min after high intensity interval running and cycling in adult men and women [31].

In the adolescent swimmers, our post-swimming results agree with Timmons et al. [9] in that no significant changes occurred in plasma IL-6 and TNF-α from pre- to post-swimming in this age group. It is important to notice the differences between ours and their study. For example, in the study by Timmons and colleagues [9], participants were non athletes, were of younger age and they cycled for 60 min at a submaximal exercise intensity (i.e., 70% VO_2max_), which is of lower intensity and involves fewer muscle groups than impacted with our swimming protocol. Regardless of these differences, and the fact that they also supplemented participants with a 6% carbohydrate beverage or placebo, both studies concluded that the inflammatory response to exercise in children and adolescents is lower than what is observed in adults, regardless of training status. According to these authors, children’s IL-6 response to sustained, vigorous exercise tends to be approximately 50% lower than in adults, suggesting that young boys may exhibit a blunted inflammatory response following vigorous exercise [32]. This blunted response seen in boys may be related to their higher resting TNF-α values, suggesting a ceiling effect that requires further research into the causal influences and mechanisms, especially during growth.

The blunted inflammatory response to vigorous exercise in children could also be a protective mechanism during growth. However, we did not measure IGF-1 or other circulating growth factors to support this speculation. A study by Nemet et al. [10] reported significant post-exercise increases in serum IL-1β, IL-6 and TNF-α compared to pre-exercise in adolescent wrestlers (16.5 ± 0.5 years old) following a standard 1.5 h wrestling practice. Significant increases from pre- to post-exercise in IL-6 but a decrease in TNF-α, were also found in non-athletic adolescent boys and girls (14.1 ± 0.8 years old) following a stationary bicycle exercise at ~80% of VO_2max_ [11]. However, it should be noted that this study did not adjust for exercise-induced changes in plasma volume, which may have impacted their results due to effects of hemoconcentration following exercise [33,34]. In addition, our results of a robust increase in IL-10 concentration immediately following high intensity swimming conflict with a previous study in healthy, non-athletic children reporting no changes in IL-10 following 1.5 h of soccer practice [12]. It is possible, therefore, that the differences in the exercise response among pediatric studies could also be attributed to their differences in mode of exercise employed (swimming, wrestling, cycling, sprinting), as well the relative intensity and duration of the exercise.

In terms of irisin, we observed a small, but significant, decrease in adults with no significant changes in adolescents following the intense swimming trial. These results are difficult to explain. One explanation could be related to the mode of the exercise being performed. Two previous studies in children and adolescents reported increases in irisin immediately following running and cycling [20,21] while the only other high-intensity swimming study in younger boys and girls found no changes in irisin immediately after each of two repeated 4 × 50 m swimming sets [35]. Alternatively, the null findings in our group of boys for irisin may be due to the participants being trained swimmers, and therefore, accustomed to the repeated bouts of swimming. In naïve, adult men, chronic exercise training has been found to result in downregulation of irisin [36], which can also explain the small post-swimming decrease in our adult swimmers, who have been involved with swimming longer. However, previous studies have typically reported increases in irisin levels immediately after different exercise protocols in men [17,19,21]. A recent meta-analysis of the effect of acute bouts of exercise on irisin levels in adults found that the overall magnitude of exercise-induced increase in irisin across studies being around 15% [37]. However, no studies in the meta-analysis included athletes.

The main strength of this study is the consideration of post-exercise plasma volume changes that adds rigour to the experimental design. The interpretation of any post-exercise result is conditional upon establishing whether exercise is effective in eliciting a response beyond the effects of hemoconcentration. A limitation to this current study is its small sample size, which, although similar to that of previous studies on the same topic, may have limited the power to detect significant changes. Another limitation of the study is that the findings are only applicable to the mode of exercise used, i.e., swimming, and may not be applicable to other forms of land-based overground activity such as running. Indeed, it is possible that a land-based activity that is predominantly eccentric and high impact, and as such applies an overall higher stress to the musculoskeletal system, may result to a greater cytokine response across ages. Future studies should consider comparing swimming with equivalent overground land-based running in both male and female athletes for a more complete picture of the inflammatory response to intense exercise.

In summary, a high intensity interval swimming trial did not induce significant acute increases in inflammatory cytokines, with the exception of anti-inflammatory IL-10, in adolescent male swimmers, while all cytokines, including IL-1β, IL-6, IL-10 and TNF-α, significantly increased in adult swimmers following the same swimming trial. Likewise, irisin levels did not significantly change in adolescent swimmers, whereas in adult swimmers the levels significantly decreased following high intensity swimming. Our results demonstrate that adolescent athletes display a blunted inflammatory response in contrast to what usually follows exercise in adults. It is possible that training-induced adaptations in adolescents follow a different timescale compared with adults. These results provide a mechanistic insight as to why young athletes require less time to recover from intense exercise and suggest that a different recovery strategy may be effective in this group.

## Figures and Tables

**Table 1:** Participant physical and training characteristics (values are mean ± standard deviation).

Variable	Adolescent Swimmers (*n* = 16)	Adult Swimmers (*n* = 16)
Age (y)	14.0 ± 1.0	21.5 ± 3.1 *
Somatic maturity (y from age of peak height velocity)	0.6 ± 1.0	n/a
Height (cm)	170.0 ± 9.3	179.6 ± 7.4 *
Mass (kg)	56.5 ± 10.5	76.7 ± 10.7 *
Body Fat (%)	11.7 ± 3.7	14.1 ± 5.2
Training History (y)	4.7 ± 1.3	9.1 ± 3.7 *
Training Volume (h/wk)	13.7 ± 0.9	15.0 ± 1.3

* denotes a significant difference between groups (*p* < 0.05).

**Table 2:** Resting (pre-swimming) circulating levels of inflammatory cytokines and myokines in adolescent and adult swimmers.

Cytokine	Group	Mean ± SE	Difference
IL-1β (pg/mL)	Adolescents	9.81 ± 5.47	*p* = 0.76
	Adults	8.32 ± 6.27	
IL-6 (pg/mL)	Adolescents	28.29 ± 9.60	*p* = 0.16
	Adults	14.94 ± 6.70	
IL-10 (pg/mL)	Adolescents	12.62 ± 1.61	*p* = 0.88
	Adults	14.79 ± 3.82	
TNF-α (pg/mL)	Adolescents	15.56 ± 1.19	*p* = 0.02
	Adults	10.43 ± 0.94	
Irisin (ng/mL)	Adolescents	13.23 ± 0.97	*p* = 0.45
	Adults	14.49 ± 1.31	

IL-1β = interleukin 1-beta; IL-6 = interleukin 6; IL-10 = interleukin 10; TNF-α = tumor necrosis factor alpha.
